# Hepatitis C seropositivity among newly incarcerated prisoners in Estonia: data analysis of electronic health records from 2014 to 2015

**DOI:** 10.1186/s12879-018-3242-2

**Published:** 2018-07-21

**Authors:** Kristel Kivimets, Anneli Uusküla, Jeffrey V. Lazarus, Kristi Ott

**Affiliations:** 1 National Institute for Health Development, 42, 11619 Tallinn, Hiiu Estonia; 2 Department of Family Medicine and Public Health, University of Tartu, Ravila 19, 50411 Tartu, Estonia; 3 Barcelona Institute for Global Health (ISGlobal), Hospital Clínic, University of Barcelona, Carrer de Casanova, 143, 08036 Barcelona, Spain; 4 CHIP, Rigshospitalet, University of Copenhagen, Blegdamsvej 9, DK-2100 Copenhagen, Denmark; 5 West Tallinn Central Hospital, Infectious Diseases Clinic, Paldiski maantee 68, 10617 Tallinn, Estonia

**Keywords:** Prison health, Viral hepatitis, Hepatitis C, HIV, Case finding, Estonia

## Abstract

**Background:**

Hepatitis C virus (HCV) infection is a widespread problem in prisons. The present study aimed to assess the prevalence of HCV seropositivity, HCV genotypes, factors associated with HCV seropositivity in newly incarcerated prisoners and to report experiences of treatment with pegylated interferon/ribavirin for HCV-positive inmates.

**Methods:**

Patient data were extracted from the Estonian prison medical information system (*Vanglate meditsiiniline infosüsteem*) databases.

**Results:**

Among 1845 prisoners newly incarcerated from January 2014 to January 2015, the overall prevalence of HCV was 56.3% (95% CI: 54 to 59), and 25.5% (95% CI: 23.5 to 27.6%) had HIV (39.0% had neither). The all-inclusive HCV testing strategy identified 37.7% more HCV infected prisoners than the risk-based (drug use history, HIV status) case finding. Factors associated with HCV seropositivity included history of drug use (aOR 6.51 95%CI 5.12–8.28), HIV co-infection (aOR 2.56 95%CI 1.92–3.43), previous incarceration (aOR 3.61 95%CI 2.48–4.04), and increasing age. The main HCV genotypes were 3a (*n* = 172, 44.4%) and 1b (*n* = 135, 35.2%). Twenty-five prisoners received HCV treatment: 60% (*n* = 15) were cured, 16% (*n* = 4) relapsed (3 with genotype 3a, one with 1b), and 12% (*n* = 3) were unresponsive (all with genotype 3a).

**Conclusions:**

HCV seropositivity rate is high and HCV tretment rate is very low in Estonian prisons. Optimizing case finding and scaling up treatment is critical to addressing the health needs of prisoners and meeting public health goals.

## Background

Hepatitis C virus (HCV) infection is an escalating global health issue and prisoners are emerging as an important high-risk group. HCV prevalence in this subpopulation ranges from 3 to 38% [1, 2]. Although this high burden of HCV is gaining notoriety and modern treatment can provide noticeable improvements in the health statuses of most patients, many countries still lack HCV prevalence data for high-risk groups. Epidemic patterns of HCV infection related to injection drug use persist in Europe. Understanding the risk factors for HCV infection among prisoners may inform HCV prevention and treatment intervention measures [2–5].

HCV prevalence is higher among prisoners than in the general population [2]. Intravenous drug use (IDU) is one of the major risk factors for HCV infection. The prevalence of IDU in prisons is ranging from 2 to 38% in Europe while the relevant percentage in the general population is estimated at 0.3% in the European Union and 0.2% in Australia. [6]. A metaanalysis showed that people who inject drugs (PWID) were about 24 times more likely to be infected with HCV than individuals in the general population in Europe [7]. IDU [8] and tattooing [9, 10] are the main risk behaviours for acquiring HCV infection in prisons.

Prisoners with human immunodeficiency virus (HIV) are more likely to be infected with HCV probably because of the similarity in the routes of transmission of these blood-borne infections [1, 3]. According to recent studies in Italian and US prisons, the HCV prevalence was more than four times higher among HCV-HIV coinfected patients than among those not infected with HIV (65.5–89.6% vs. 15.5–27.5%, respectively) [3, 11].

Another risk factor related to HCV infection is repeated incarceration [12, 13], an association that may be confounded by low socioeconomic status, poor access to health care, high-risk sexual behaviors and injection drug use [14].

In some studies HCV infection was observed more frequently in female inmates than in males. [1]. In part that may have been because the number of women in prison has grown and women prisoners are more likely than their male counterparts to have been incarcerated for drug offences [15] and to have serious drug-related health problems [16], including infectious diseases [17].

Direct-acting antivirals (DAAs) are relatively new and expensive, but cost-effective when well utilised as interventions for chronic hepatitis C, and preliminary results suggest that DAAs may eradicate HCV from the blood (sustained virological response). The effect of long-term treatment with DAA (hepatitis C morbidity and mortality) is yet to be convincingly demonstrated [18]. Still, ribavirin has been an integral component of treatment for hepatitis C virus (HCV) infection, and the safety profile of ribavirin is improved when co-administered with all-oral DAA combinations in the absence of interferon [19]. Several studies have shown that prisoners can be effectively treated for HCV infection [20–22], although treatment is administered less frequently to prisoners because of the cost of treatment, treatment often exceeding average prison stay combined with a lack of continuity of care, and unawareness of the HCV infected status of the prisoners [1].

Most HCV infected people are unaware of their condition [23]. Prisons may be a good venue to detect many of those cases. Although exact data are not available, each year, up to one million people with undiagnosed HCV infection may come into contact with the correctional system in the United States [24]. Strategies of case finding should be developed that are based on current infections rates and feasibility.

### The situation in Estonian prisons

The prison population in Estonia has stabilised at nearly 3000 individuals in recent years but the incarceration rate, with 222 prisoners per 100,000 population, still remains very high. According to the Council of Europe’s annual penal statistics, the highest incarceration rates in Europe are recorded in the former Soviet Union countries (439 per 100,000 population in the Russian Federation, 277 per 100,000 in Lithania, 274 per 100,000 in Georgia, 248 per 100,00 in Azerbaijan and 223 per 100,000 in Latvia) [25].

Needle and syringe exchange programmes (NSP) in Estonia were launched in 1997. In addition to NSP, condom distribution, different counselling services incl. social, health, psychological, legal, experience counselling), basic humanitarian aid, primary hygiene possibilities [26], naloxone take-home program training (since 2013), and motivation and referral to treatment/rehabilitation are offered under harm reduction services in Estonia. From 2015, naloxone is available also in prisons [27]. In all Estonian prisons, opioid substitution therapy (OST) in the form of methadone treatment is provided. NSP are not operating in Estonian prisons [28].

According to the Estonian Ministry of Justice, there were 3580 prisoners in Estonia’s four prisons during the period between 1 January 2014 and 1 January 2015 (personal communication with Asko Oruste, the information systems manager with Estonian IT Support System). A study conducted in Estonia in 2012–2013 found that, of all prisoners, one-third had a history of drug use, 15.6% were infected with HIV and, of those that were HIV-positive, 54.1% were receiving antiretroviral therapy (ART) [28].

The implementation of the project “Improvement of Prevention and Treatment of Infectious Diseases in Estonian prisons” [29] targeting testing, treatment and infrastructure buildings was a milestone in the diagnosis and care of HCV in prisons [30]. Before that, HCV testing targeted only prisoners considered at high risk for HCV (identified by their drug use history or HIV status; “the old testing strategy”) (personal communication with Maret Miljan from the rehabilitation department of the Estonian prison department,). Since the launch of the project in January 2014, HCV tests have been offered for all newly incarcerated prisoners (“the new testing strategy”).

The aim of this study was to assess the prevalence of HCV seropositivity and its related factors among newly incarcerated prisoners and to describe the effects of treatment of HCV infection in Estonian prisons.

## Methods

This study was carried out from 1 January 2014 to 1 January 2015. The term ‘newly incarcerated prisoner’ is used for all people, including adults and juveniles, detained during the investigation of a crime, while awaiting trial, after conviction, before sentencing and after sentencing, and incarcerated in one of the four Estonian prisons.

### Data and data sources

For this study we relied on the secondary data available in the administrative and health databases of the Estonian prison medical information system.

#### Prison electronic patient database

The prison electronic patient database is designed for prison healthcare and contains electronic health records. For those tested for HCV, the following data were extracted from prisoners’ health records: (i) HCV test result, (ii) HCV genotype, (iii) age, (iv) gender, (v) HIV serostatus, (vi) history of drug use (based on the International Classification of Diseases codes F11-F16 or F18-F19), and (vii) HCV treatment results. HCV in prisons is treated by prison infectious diseases physicians who also make HCV-genotype testing decisions.

For all samples collected in the prison medical information system over the study period, HIV testing (VironostikaHIV Uniform II Ag/Ab, BioMerieux method), anti-HCV antibodies testing (Siemens, A HCV200T KIT), and HCV genotyping (The VERSANT® HCV Genotype 2.0 Assay) were performed at Synlab Estonia (an accredited laboratory following the requirements of ISO 15189 Medical Laboratories).

#### Database of prisoners, detained persons, persons in custody and probationers

Data on tattoos and previous incarceration were extracted from the databases of prisoners, detained persons, persons in custody and probationers (hereinafter referred to as the *prisoners’ register*). The prisoners’ register is an electronic database containing selected information on the details of prison sentence as well as personal data. Information on tattoos included in the register is based on inspection of prisoners’ skin at prison entry.

### Statistical analysis

Descriptive statistics are presented for categorical variables (absolute number (n) and relative frequencies (%)) and continuous variables (mean, standard deviation and range). A chi-squared test was used to explore differences in categorical variables and the Wilcoxon rank sum test for age comparisons between the HCV infected and non-infected groups. Associations between antibodies to HCV (the outcome of interest) and covariables were explored bivariably and multivariably (by including all independent variables in the model) by logistic regression analyses. Odds ratios (OR) and adjusted OR (AOR) together with 95% confidence intervals (CI) are presented. Associations between covariables were also tested to assess the reliability of the multivariate models (according to the possibility of multicollinearity). The statistical software program SPSS version 16.0 (Chicago, Illinois, USA) was used.

## Results

### HCV and HIV diagnosis

Between 1 January 2014 and 1 January 2015, 1957 new prisoners were admitted to the Estonian prison system. Of those, 603 were already infected with HCV (based on data from previous testing in prison). As per the new testing strategy, the rest of the newly imprisoned individuals (*n* = 1242) were tested for HCV with the exception of 112 prisoners (26 prisoners declined testing and the reason for not testing was unknown for the other 86).

The final sample consisted of 1845 newly incarcerated prisoners with known HCV status. They had an average age of 35 years (SD = 11.54, range 15–78). Of these prisoners, 94.4% were male (*n* = 1742), 58.1% had previously used illicit drugs (*n* = 1034), 28.1% were tattooed (*n* = 519), and 68.9% had been previously incarcerated (*n* = 1272). The prevalence of HCV seropositivity was 56.3% (*n* = 1038, 603 + 435) and the prevalence of HIV 25.5% (95% CI 23.5–27.6%) (*n* = 469). One fifth (*n* = 379; 20.5, 95% CI 19.8–23.6%) of the newly incarcerated prisoners had HCV-HIV co-infections. The majority (80.8, 95% CI 77.2–84.4%) of the HIV-positive prisoners were also infected with HCV (Table 1).

**Table 1:** Factors associated with hepatitis C infection among newly incarcerated prisoners in Estonia, 2014 to 2015 (*n* = 1845)

Variable	All *N* = 1845	HCV + ^b^ (n; %)	HCV-^c^ (n; %)	*p*-value (HCV + vs HCV-)
Age (mean; SD)	34.1; 10.92	35.66; 11.49	34.11; 11.56	*p* < 0.001
Gender (male: n; %)	1742; 94.4%	978; 56.1%	764; 43.9%	*p* = 0.675
Age group				
15–20	107	36; 33.6%	71; 66.4%	
21–30	620	330; 53.2%	290; 46.8%	
31–40	494	304; 61.5%	190; 38.5%	
41–50	304	177; 58.2%	127; 41.8%	
51–60	183	103; 56.3%	80; 43.7%	
61–70	30	25; 83.3%	5; 16.7%	
71–78	4	3; 75%	1; 25%	
Tattoo (yes: n; %)	519; 28.1%	331; 63.8%	188; 36.2%	*p* < 0.001
Drug use history (yes: n; %)	1034; 56.2%	819; 76.6%	250; 23.4%	*p* < 0.001
HIV^a^ infected (n; %)	469; 25.5%	379; 80.8%	90; 19.2%	*p* < 0.001
Previous incarceration (yes: n; %)	1271; 68.9%	827; 65.1%	444; 34.9%	*p* < 0.001

^a^HIV: human immunodeficiency virus

^b^HCV**+**: Hepatitis C virus positive

^c^HCV**-**: Hepatitis C virus negative

The old testing strategy – high-risk based case finding - would have identified only 37.7% (164/435) of the HCV infected newly incarcerated prisoners.While comparing HCV infected and non-infected newly incarcerated prisoners, we found that HCV-infected prisoners tended to be slightly older, and were more likely to have a tattoo (63.8% vs 36.2%, *p* < 0.001), to have a history of drug use (76.6% vs 23.4%; *p* < 0.001), to be HIV-infected (80% vs 19.2%, *p* < 0.001), and to have previously been incarcerated (65.1% vs 4.9%, *p* < 0.001). There were no gender differences in HCV seropositivity (Table 2).

**Table 2:** Factors associated with hepatitis C infection among newly admitted prisoners in Estonia, 2014 to 2015, univariate and multivariable logistic regression analyses (*n* = 1845)

Variable	Bivariable analysis		Multivariable analysis	
	OR ^c^	CI^b^ (95%)	Adjusted OR	CI (95%)
Age	1.01	1.00–1.02***	1.01	1.00–1.02***
Gender (female)	1.09	0.73–1.63	1.30	0.79–2.15
Tattoo (yes)	1.54	1.25–1.90***	0.81	0.62–1.04
Drug use history (yes)	8.49	6.87–10.49***	6.51	5.12–8.28***
HIV^a^ infected (yes)	4.62	3.59–5.96***	2.56	1.92–3.43***
Previous incarceration (yes)	3.2	2.61–3.93***	3.16	2.48–4.04***

*** *p*-value < 0.01

After adjustment, history of drug use emerged as the factor most strongly associated with HCV seropositivity (AOR 6.51 95% CI 5.12–8.28). The risk of HCV seropositivity was also significantly higher among those who were HIV-positive (AOR 2.56 95% CI 1.92–3.43) and those previously incarcerated (AOR 3.16 95% CI 2.48–4.04). The risk of HCV positivity increased with age. While tattooing was associated with the HCV rates in the bivariable analysis, this association was not found in the multivariable analysis.

### HCV genotypes

Of the 1038 prisoners who tested positive for HCV, 383 (36.9%) underwent HCV genotype testing. The main genotypes identified were 3a (44.4%, *n* = 172) and 1b (35.2%, *n* = 135). A further 17% (*n* = 65) had genotype 1a, and 2.6% prisoners (*n* = 10) had genotype 2. Only one prisoner had HCV genotype 4.

### HCV treatment

Between 1 January 2014 and 1 January 2015, all HCV cases, 25 prisoners (24 male and 1 female), received HCV treatment (pegylated interferon (PEG-INF) and ribavirin). The decision to treat was reached after an in-depth, individualized process including informed consent and considering other factors such as co-morbid addiction and mental illnesses. For patients with a history of drug use, a program-based addiction treatment [31] was required to initiate HCV treatment. Inmates who had a history of depression or another psychiatric disorder were referred to an on-site psychiatrist for evaluation. Prisoners with HCV genotype 1b or with HIV infection were treated for 48 weeks, and those with genotypes 2 or 3 were treated for 24 weeks [32]. Individual treatment decisions were made by prison infectious disease physicians based on whether the expected length of stay was long enough to allow for the completion of a treatment course (24 to 48 weeks). Clinical indicators for treatment and funds available for the medications were also taken into consideration. Of those who received HCV treatment, 21 (84%) had a history of drug use (11 opiate users, 5 stimulant users and 5 users of multiple drugs including opiates), 5 (20%) were HIV infected, none was hepatitis B infected, 24 (96%) had received hepatitis B vaccination before treatment.

None of the patients had previous experience with interferon and ribavirin. The average age of treated patients was 35.5 years (range 29–45). Fifteen of 25 (60%) treated patients underwent ultrasound-guided liver biopsies. The Metavir scoring system was used to classify fibrosis levels [33]. 11 (91.7%) of those biopsied had no to mild fibrosis, 1 person had moderate (9%) and 3 (27.3%) had severe fibrosis of the liver. Of all the patients treated, 15 (60%) were cured based on a negative HCV RNA test at the end of the treatment and 6 months after the treatment; 4 (16%) relapsed based on a negative HCV RNA at the end of the treatment and positive HCV RNA test 6 months after the treatment (3 patients with genotype 3a, one with 1b); 3 (12%) were unresponsive to treatment based on an HCV RNA test at 24 weeks of treatment (all with genotype 3a); 1 person (4%) stopped treatment; and treatment outcome data were missing for 2 (8%) of the patients as they had been released from prison.

## Discussion

To our knowledge, this is the first study focusing on HCV prevalence and HCV treatment outcomes in the entire prison population of a European country. The studies that do exist are often restricted to individual prisons or subgroups of prisoners [2].

We found high HCV seropositivity rates (56%) among newly imprisoned individuals in Estonia. HCV seropositivity among newly incarcerated and tested prisoners (with either unknown HCV status or previous negative HCV test) was 35%. These prevalence figures are consistent with those from Italy (38%) [34] and Scotland (19%) [35], where available data suggest that HCV is highly prevalent among prisoners.

The risk factors for HCV seropositivity we observed (drug use, previous incarceration, HIVstatus, age) are in accordance with previous research [1, 2, 8, 14, 36]. According to previous studies in other countries, the main risk factor associated with HCV infection in prison populations is IDU [1, 2, 8, 37]. In our study, the odds for being HCV-positive were about three times higher among HIV-positive than HIV-negative prisoners. HIV and HCV share common routes of transmission, and are therefore expected to some extent co-infect the same individuals.

Previous incarceration has been significantly associated with HCV infection in studies from Australia, England, and Wales [36–38]. Multiple incarcerations were also found to be a risk factor for HCV seropositivity in our study. The contribution of HCV transmission in prison to the overall disease burden among PWID is difficult to document owing to uncertainties regarding precise date of infection and risk behaviours in the community (both prior to and post-incarceration). The role of prisons as a risk environment for the reinforcement, concentration and amplification of infections such as HIV and HCV has been suggested [39].

In our study, the seropositivity of HCV was higher among those with tattoos. However, this association was lost while adjusting for potential confounders. It is known that tattooing is common in prisons and jails [10] and prisoners who acquired their tattoos in prisons are more likely to be HCV-infected [1, 12, 34]. The risks and benefits of legal tattooing in prison should be discussed. Evidence from studies on the relationship between tattooing and HCV transmission in prisons appears to be mixed. Some studies report significant correlations [10, 36, 40] while others do not [41].

We found the presence of hepatitis C antibodies in 12 individuals (0.6%) who were HIV negative and had no records indicative of drug use. While we do not have data on other HCV risk factors (such as history of blood transfusion, high risk sexual behviour), all of the 12 prisoners had tattoos. Eight of them had acquired their tattoos while in prison during the Soviet era (personal communication with prisoners). One might speculate that the traditions of tattooing in prisons have changed (there appear to be fewer tattoos among more recent prison populations and drug using individuals).

Data on the relationship between HCV and gender in prison populations is also conflicting. In some studies, HCV infection was observed more frequently in incarcerated women than in incarcerated men. [1, 3]. Other studies did not find the same result [42]. In our study, after accounting for other risk factors, gender did not emerge to be associated with HCV seropositivity.

Screening strategies based on risk factors have traditionally failed to identify all patients at risk of infection, leaving a sizable proportion of patients unaware of their infection. We observed that a screening system for HCV case finding relying only on known risk factors misses a sizeable proportion of newly incarcerated individuals with HCV infection. The all-inclusive HCV testing strategy identified 37.7% more HCV infection cases among the newly incarcerated than would have been detected on the basis of known risk factors (drug use history, HIV infected status) screening.

In our study, the main HCV genotypes were 3a (*n* = 172, 44%) and 1b (*n* = 135, 35.2%). HCV genotypes have geographically distinct distributions. HCV genotype 1 and 3 infections are globally more prevalent than all other genotypes. Those genotypes, more commonly found in lower-income countries, account for a significant proportion of HCV cases worldwide [43]. Meyer et al. found that genotype 3 prevailed in prisoners from the independent states of the former Soviet Union [44]. Tyczyno et al. compared the HCV genotyping performed on Polish prisoners and hospital patients, and found that HCV genotype 3 prevailed in the prisoners (60.1%) and genotype 1 in patients at large (79.6%). This distinction is most probably due to the fact that genotype 3 is more frequent in PWID [45]. In Estonia, the following distribution of genotypes was documented among hospital patients: genotype 1b was found in 32% of the patients, 3a was found in 20%, 2a in 12%, and 1a/b (double infection) in 28% [46]. Among the Estonian prisoners tested for HCV genotypes nearly two decades later, we were unable to replicate the finding of 1a/b double infection occurrence.

The response to antiviral treatment in prisoners is similar to that of the general population [1]. Our study observed a 60% sustained viral response among treated prisoners. Importantly, this result does not differ from the treatment response reported from the community (in a hospital setting in Estonia) where a 60.3% sustained treatment response was achieved [47]. This suggests that acceptable HCV treatment outcomes can be achieved in prisons. There are clear advantages to HCV treatment in prison settings. In-prison, treatment provides the opportunity to access population groups for whom treatment options may be limited after release. Treatment in prisons occurs in controlled, directly observed settings, potentially achieving increased compliance and monitoring for side effects and complications [48, 49].

Our study has some strengths and limitations. A key feature of our study is the all-inclusive testing strategy and national coverage of the data collection. This is different from other, similar studies [11, 12, 14] conducted in the region because testing was offered and data were collected from all new prisoners entering all Estonian prisons. However, given the nature of the data used, we were unable to determine the frequency or duration of drug injection and, particularly importantly, distinguish between injecting and other types of drug use. Thus, some of the important details might have been lost. Importantly, we were unable to assess the impact of other potential HCV risk factors (such as sexual behaviour and previous blood transfusions, or surgery).

## Conclusions

Case finding for HCV infection in prisons, HCV treatment and educational programmes to discourage high-risk behaviours might help curtail the rise in HCV infection in prison populations. Our study suggests that HCV treatment in infected inmates is feasible and the results (sustained viral response achievement) do not differ from that in the community.

Reducing HCV prevalence among prisoners has the potential to reduce the burden of disease in the general public in the community. When developing HCV screening strategies in prisons, policy-makers should consider the risk factors that we have highlighted in our study.

Our findings have several implications: they show that (i) universal HCV testing at prisonentry has the potential to increase the number of people who are aware of their infection, (ii) there is a need to scale up comprehensive HCV care to those infected. We believe that the results of this study might be applicable to other countries in the region that have witnessed injection drug use and related HIV epidemics in the late 1990s and early 2000s.

## Abbreviations

AOR: Adjusted odds ratio
ART: Antiretroviral therapy
CI: Confidence interval
DAA: Direct-acting antivirals
HCV: Hepatitis C virus
HIV: Human immunodeficiency virus
IDU: Injection drug use
NSP: Needle and syringe exchange programmes
OR: Odds ratio
OST: Opioid substitution therapy
PWID: People who inject drugs
